# Beyond balance: the impact of adult sex ratios on reproduction and longevity in *Zophobas morio* rearing

**DOI:** 10.1007/s10522-024-10178-8

**Published:** 2025-01-08

**Authors:** P. Soulioti, C. Adamaki-Sotiraki, C. I. Rumbos, C. G. Athanassiou

**Affiliations:** 1 Department of Agriculture, Laboratory of Entomology and Agricultural Zoology, Crop Production and Rural Environment, University of Thessaly, Phytokou Str., 38446 Volos, Greece; 2 Department of Agriculture, University of Patras, 30200 Messolonghi, Greece

**Keywords:** Adult sex ratio, Longevity, Fecundity, Fertility

## Abstract

Over the years, the study of adult sex ratio is a topic that has gained attention for its impact on reproductive outcomes and aging across various insect species. However, there is still limited research focused on insects reared for food and feed production. To address this gap, this study aimed to evaluate the impact of different adult sex ratios on the reproductive dynamics and longevity of the superworm, *Zophobas morio* (F.) (Coleoptera: Tenebrionidae), a species with interesting potential as a nutrient source. In this study, we assessed three adult sex ratios, i.e., 5:5, 6:4 and 8:2 (female:male) with mesh used to each setup. An additional 5:5 sex ratio without mesh served as control. On a weekly basis, adult survival, egg production, and egg hatching rates were recorded. Our results revealed statistically significant differences in egg production across the different sex ratios tested, but no statistically significant differences in hatching rates and adult survival. These findings suggest that both balanced and female-biased sex ratios are suitable for *Z. morio* and that it is important to consider this factor when enhancing efficiency in large-scale insect production.

**Graphical abstract:**

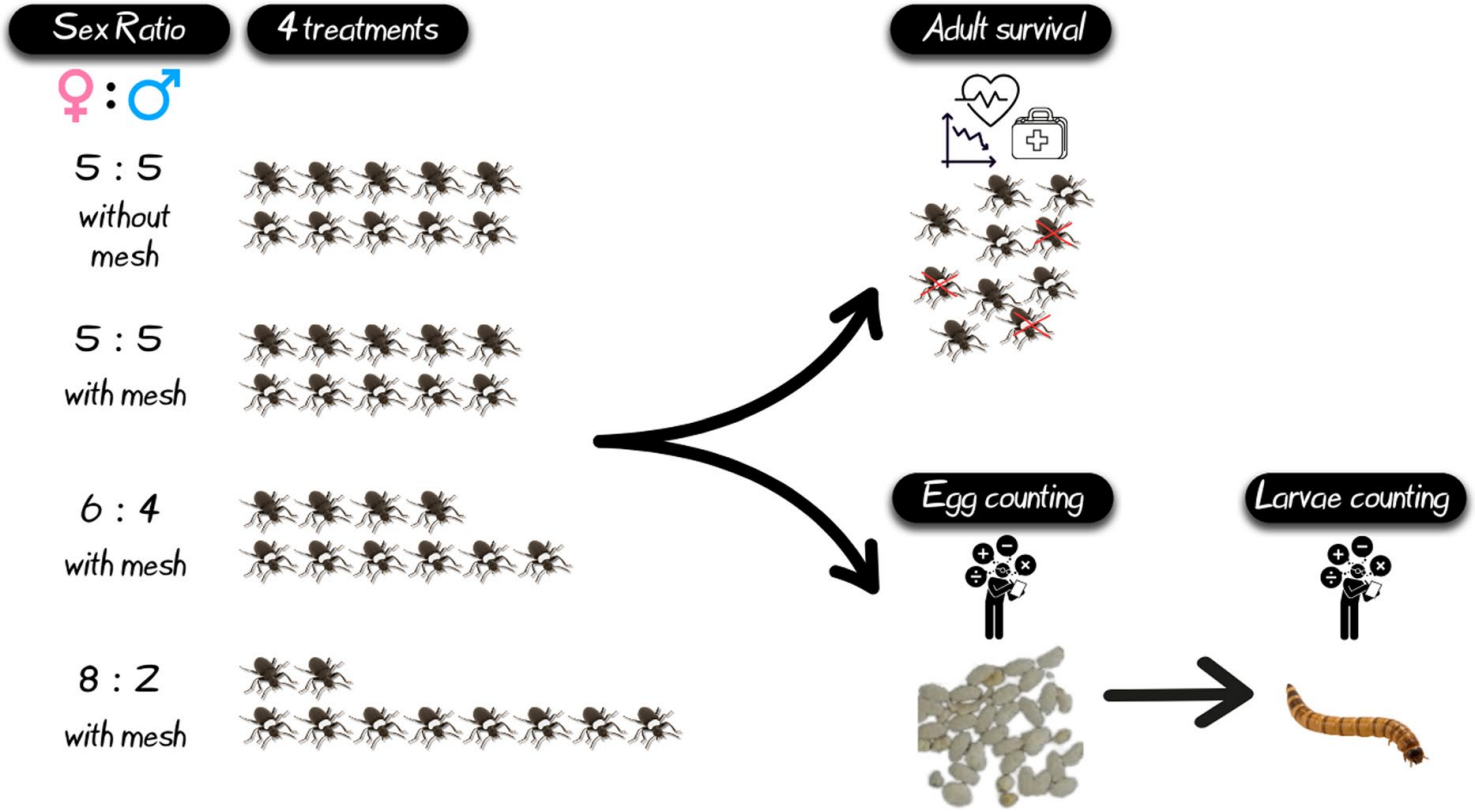

## Introduction

The insect farming sector has experienced rapid global development only in the last decade, although the use of insects as food and feed was suggested much earlier (Meyer-Rochow 1974; Mancini et al. 2022). Insects are a promising and sustainable source of protein, offering a viable solution to the growing demand for food arising from the increasing global population (Van Huis et al. 2013; FAO 2019). However, to fully harness insects as a sustainable food source, large-scale and efficient insect farming is necessary (Lange and Nakamura 2021). Considerable research has been focused on the optimal feeding substrates, population density, environmental conditions (such as humidity, temperature, photoperiod etc.) and the mating compatibility between different strains of the same insect species (Riekkinen et al. 2022; Van Broekhoven et al. 2015; Rumbos et al 2020; Zafeiriadis et al. 2023; Kwak et al. 2021; Adamaki-Sotiraki et al. 2022a). Nevertheless, sex ratio is a crucial factor influencing insect reproductive dynamics, and further data are needed to optimize rearing practices and unlock the full potential of insects for food and feed production.

One critical aspect of optimizing insect reproduction is understanding the strategies species employ to maximize their reproductive output and their fitness in general (Torres-Vila 2012). A key factor influencing reproductive dynamics is the ratio of reproductive females to males (Wehbe and Shackelford 2021). In most species, the sex ratio tends to be 1:1, in line with Fisher’s principle (Sedláková et al. 2022; Papach et al. 2019). However, some species exhibit male- or female-biased sex ratios (Papach et al. 2019). These imbalances can significantly affect the mating opportunities (Kageyama et al. 2003; Atlan et al. 2004; Cheng et al. 2023), with female-biased ratios observed in species like *Cnaohalocrocis medinalis* (Lepidoptera: Pyralidae) (Guo et al. 2019) and male-biased ratios in species like *Apis* sp. (Page and Metcalf 1984). While the sex ratio is a crucial biological factor affecting the population growth of arthropods (Cheng et al. 2023), its impact on the reproductive output of insects has not been studied thoroughly yet. Recent findings suggest that sex ratio affects individual reproductive effort, with potential consequences for somatic maintenance (Rodríguez-Muñoz et al. 2019; Tompkins and Anderson 2019) and pivotal trades-off between reproduction and longevity (Partridge and Barton 1993; Kirkwood and Holliday 1979; Jehan et al. 2020). Therefore, manipulating sex ratios could be an important tool to enhance reproduction in artificially reared insect populations.

*Zophobas morio* (F.) (Coleoptera: Tenebrionidae), commonly known as giant mealworm or superworm, is a large black beetle native to Central and South America (Tschinkel 1984). Its larvae can grow up to 55 mm in length and weigh between 0.8 and 1.2 g (Tschinkel 1993; Friederich and Volland 2004). It belongs to the Tenebrionidae family, which includes other species like the yellow mealworm, *Tenebrio molitor* L. (Coleopter: Tenebrionidae), and the lesser mealworm, *Alphitobius diaperinus* (Panzer) (Coleoptera: Tenebrionidae), and the two species of mealworms are approved for use as food and feed in Europe (Rumbos and Athanassiou 2021; Commission Regulations (EU) 2021/1975; 2022/188; 2023/58]. Traditionally used as feed for pets such as small mammals, reptiles and birds (Jabir et al. 2012; Park et al. 2013), *Z. morio* also holds a great potential as a food resource due to its high nutritional value, with protein content ranging from 45.3 to 68.05 g per 100 g of dry matter (Soares Araújo et al. 2019; Ribeiro et al. 2019; Kulma et al. 2020). Countries like Australia have already approved the consumption of *Z. morio* as food, while Insect Protein Association of Australia (IPAA) provides guidance to stakeholders (IPAA 2023). More recently, Singapore’s Food Agency (SFA) has followed other countries steps, including European Union, New Zealand, Thailand, South Korea and Australia in authorizing the consumption of 16 insect species, including *Z. morio* (SFA 2023; 2024).

Despite Food and Agriculture Organization of the United Nations (FAO) actively promotes the rearing of edible insects, the optimization of rearing practices for *Z. morio* still remains largely unexplored. While recent studies have focused on optimal rearing practices, the influence of adult sex ratio on reproductive output and longevity has yet to be explored. Given this knowledge gap and the recognized importance of sex ratio in species reproduction, this study aims to evaluate how the different sex ratios in *Z. morio* populations affect fecundity, fertility and longevity.

## Materials and methods

### Insects

Individuals of *Z. morio* from the colonies maintained at the Laboratory of Entomology and Agricultural Zoology at the University of Thessaly were used for experimentation. Larvae were reared on wheat bran received from a local provider and were supplemented with agar (20 g/l) cubes twice a week. Due to the fact that *Z. morio* larvae fail to pupate under crowded conditions, late-instar larvae were individually placed in cylindrical plastic containers (85 × 30 mm) with a 15 mm opening on the lid covered with muslin gauze for pupation. After eclosion, adults were kept in plastic boxes (48 cm length × 28 cm width and 10 cm height) with a rectangular screened opening (19 × 27 cm) on the top cover to allow air circulation. Cardboard egg-packing and empty roll papers were used as shelter and breeding-places for adults. Adults were also provided with wheat bran and were left to mate and oviposit on empty paper rolls. All rearing boxes with the stock *Z. morio* colonies were kept in constant condition, i.e., 26 ± 1 °C, 55 ± 5% relative humidity (RH) and continuous darkness.

### Experimental design

**Fig. 1:**
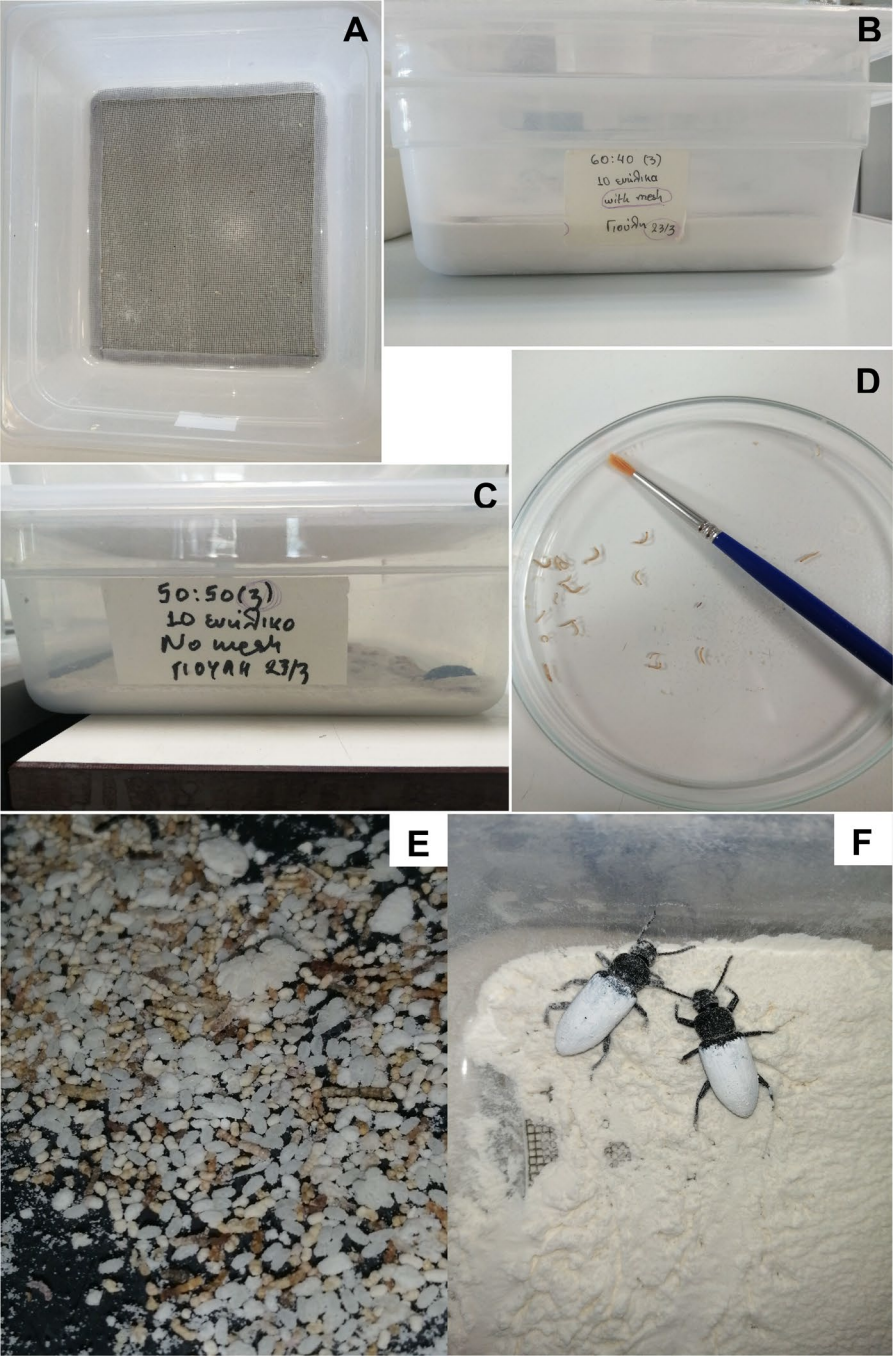
Experimental setup: **A** Oviposition box with a mesh bottom, **B** Stacked oviposition boxes with mesh, **C** Oviposition box without mesh, **D** Hatched larvae, **E** Zophobas morio eggs, **F** Females marked with white oil-based paint for identification

Initially, virgin beetles were obtained by separating females and males at the pupal stage, based on the differences in pupal abdominal appendages observed under a digital stereoscope (Levenhuk DTX RC4 Remote Controlled Microscope, Levenhuk, Inc., USA) (Barké and Davis 1967; Bhattacharya et al. 1970). After sex determination, female and male pupae were kept separately until adults’ emergence. Newly emerged, dark colored adults (7–14 d old to ensure sexual maturity) were utilized in the bioassay. Prior the bioassay, the mature female beetles were marked individually by applying a small dot of oil-based paint (Paint Marker, Medium P-520, White Color, + EFO) on the dorsal surface of the thorax. Briefly, *Z. morio* adults were gently put into a bag made of mosquito net (18 mesh). After holding gently, the mesh down around each beetle to immobilize it, a thin, soft hairbrush was used to apply a small drop of oil-based paint to the beetle (Fig. 1). Once the paint dried, groups of 10 adults were placed in the oviposition boxes at three ratios of females to males (i.e., 5:5, 6:4 and 8:2). Every group of adults (per container) was weighed before the experiments to ensure that adults of similar weight were used among treatments. As oviposition boxes, plastic containers were used, whereas wheat flour received by a local provider (particle size < 0.5 mm) was used as oviposition substrate for the adults. Briefly, two plastic containers were stuck inside another. The bottom of the upper vial was removed and replaced by a plastic mesh grid (2 × 2 mm hole diameter) to permit egg oviposition and avoid egg cannibalism by the adults (Fig. 1). The bottom container was filled with wheat bran (< 0.5 mm) as oviposition substrates up to the level of the mesh (Fig. 1). As control, a 5:5 ratio was utilized without mesh (Fig. 1). Agar (20 g/l) cubes (2 cm^3^) were provided to beetles as a moisture source three times a week, as larvae deprived of water exhibit strong cannibalistic behavior (Ichikawa and Kurauchi 2009). Beetles were left to mate and oviposit undisturbed for 7 days. Eggs were collected weekly by sieving the oviposition substrate with a 250 μm opening sieve and counting the laid eggs (Fig. 1). Sometimes females may glue their eggs to the inner part of the plastic container, instead of laying them in the oviposition substrate. Consequently, the glued eggs of the control treatment were counted together with collected eggs. Both glued eggs and eggs collected from the substrate were left in the same plastic boxes (48 cm length × 28 cm width and 10 cm height), with a small amount of wheat flour as feeding substrate for the newly emerged larvae. Adults were transferred into new containers. Collected eggs from the treatments using a mesh were placed in cylindrical vials supplemented with wheat flour, whereas adults were placed back in the oviposition boxes. The survival rate of adults was also recorded. Collected eggs were allowed a maturation period of 14 days and the egg hatching rate (number of emerged larvae) was recorded (Fig. 1). The experiment lasted 91 days and was performed under controlled rearing conditions, i.e., 26.0 ± 0.5 °C, 55 ± 5% relative humidity and continuous darkness. There were four replicates for each treatment, using separate groups of newly emerged beetles for each replicate.

### Data analysis

To estimate the cumulative number of eggs per female, the average numbers of eggs per females at each evaluation point were summed. The determination of the hatching rate was the division of the number of hatched larvae and the respective number of eggs. To define the cumulative hatching rates, the cumulative number of larvae per live female were divided by the cumulative number of eggs per live female for each treatment at each time point.

The normality of cumulative number of eggs per female, cumulative hatching rate and final survival rate of both females and males was tested using the Shapiro–Wilk test, while Levene’s test was used to evaluate homogeneity of variances (homoscedasticity). Both the cumulative number of eggs per female and cumulative hatchability met the assumptions and were analyzed with one-way ANOVA, with differences between treatments determined through Tukey’s HSD test. However, the final survival rate for both females and males did not meet the assumptions required for one-way ANOVA, so the non-parametric Kruskal–Wallis H test was applied instead. To further evaluate the interaction between female-to-male ratio and time on fecundity, hatchability and adult survival, a Linear Mixed-Effect Model were conducted to assess how these variables are affected over time. All the data were analyzed statistically by SPSS 26.0 (IBM Corporation, Armonk, NY USA).

## Results

**Fig. 2:**
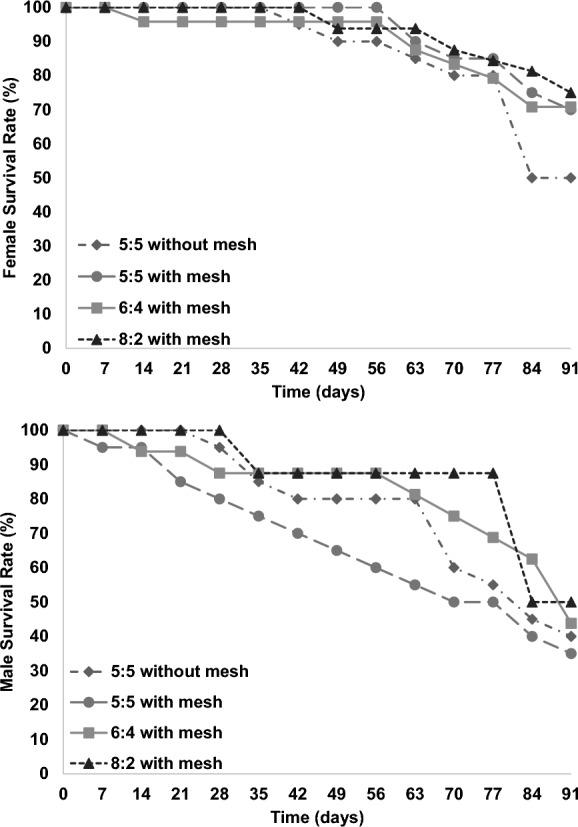
Survival rate for female and male adults of *Zophobas morio* (%, mean)

The survival rate for both female and male *Z. morio* adults decreased over time (Fig. 2). No statistically significant differences were detected for the final adult survival rate, while the linear mixed-effect model revealed that there were also no statistically significant differences regarding sex ratio in each time period (F = 2.400; df = 3, 156; P = 0.070). However, statistically significant differences were apparent for the female survival rate across the different sex ratios over time (F = 9.325; df = 12, 156; P < 0.001). In terms of interaction between sex ratio and time, it was revealed that sex ratio does not significantly affect female survival rate over time (F = 0.383; df = 36, 156; P = 0.999). Female’s survival rate remained high (> 80%) for all treatments until the 56th day. For the last two measurements a sharp decrease for the control treatment (5:5 without mesh) was recorded (50% final survival). In comparison, the 5:5, 6:4, and 8:2 with mesh treatments exhibited a slower decline in the female’s survival rate, with final survival rates ranging from 70 to 75%.

Conversely, male survival rates were lower over time comparing to those of females. Most treatments showed a gradual decline in male survival during the first 28 days, with survival rates ranging from 80 to 95%. Interestingly, the 8:2 treatment was an exception, maintaining a stable male survival rate with no decline during this initial period. For the 8:2 treatment, the male survival rate remained at a high level (88%) until the 77th day, while for the treatments 6:4 and 5:5 without mesh the survival rates exceeded 80% until the 63rd day. Consequently, a severe decline in the male survival rate of 6:4 and 5:5 without mesh was observed from the 63rd day till the completion of the experiment, while for the 8:2 treatment a sharp decrease was recorded on the 84th day. It is worth mentioning that for the 5:5 with mesh treatment the decline in male survival rate was the highest among the treatments over time. The final survival rate of males ranged from 35 to 50%. However, there were no statistically significant differences in the final survival rate, neither for the males nor for the females (Males: P = 0.938; df = 3 / Females: P = 0.313; df = 3). The linear mixed effect model analysis revealed that both sex ratio (F = 6.505; df = 3, 156; P < 0.001) and time (F = 8.391; df = 15, 156; P < 0.001) significantly affected male survival rate, while there were not statistically significantly differences in terms of interaction between sex ratio and time (F = 0.271; df = 36, 156; P = 1.000) (Table 1).

**Table 1:** Linear mixed-effect model analysis for number of eggs laid per female, hatching rate, and survival rate of *Zophobas morio* across four different sex ratio treatments (5:5 without mesh, 5:5 with mesh, 6:4 and 8:2) over a period of 91 days

Source	Numerator df	Denominator df	Eggs per female		Hatching rate %		Female survival rate %		Male survival rate%	
			F	*P*	F	*P*	F	*P*	F	*P*
Intercept	1	156	707.817	< 0.001	1105.547	< 0.001	7356.781	< 0.001	2142.223	< 0.001
Sex ratio	3	156	7.394	< 0.001	2.420	0.068	2.400	0.070	6.505	< 0.001
Time (weeks)	12	156	28.760	< 0.001	5.658	< 0.001	9.325	< 0.001	8.391	< 0.001
Interaction	36	156	1.306	0.136	0.551	0.981	0.383	0.999	0.271	1.000

**Fig. 3:**
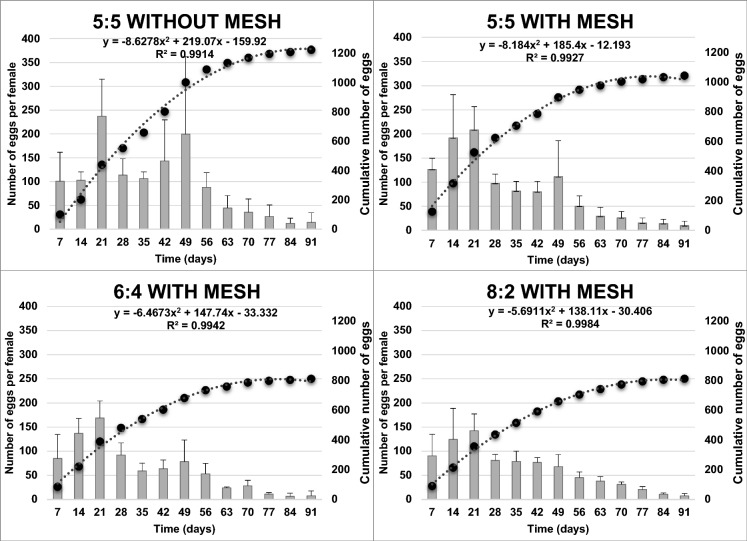
Weekly oviposition patterns of *Zophobas morio* under different adult sex ratio treatments over 91 days (mean ± SE). The bars illustrate the number of eggs per female at each time period. The dash lines indicate the fit curve for the cumulative number of eggs per female over time

The cumulative number of eggs per female over time is presented in Fig. 3. Egg production exhibited the highest number for all the treatments on the 21st day. The egg production decreased gradually over time until the termination of the experiment. However, an increase in the number of eggs per female was detected at the 49th day for the 5:5 sex ratio with and without mesh, as well as for the 6:4 sex ratio. The 8:2 sex ratio was the only exception that did not present any increase in egg production after the 21st day. Statistically significant differences were recorded in the cumulative number of eggs per female at the end of the experiment (F = 4.231; df = 3; P = 0.029). The highest cumulative number of eggs was produced for the 5:5 without mesh treatment with 1223 eggs in total. However, there were no statistically significant differences between the control treatment (5:5 without mesh—1223 eggs in total) and the 5:5 with mesh treatment (1043 eggs in total). The cumulative number of eggs per female for the 5:5 without mesh treatment was statistically higher than those of the 6:4 and 8:2 treatments (813 eggs in total). In contrast, the cumulative number of eggs across treatments with mesh did not present statistically significant differences. Based on the linear mixed effect model analysis, both sex ratio (F = 7.394; df = 3, 156; P < 0.001) and time (F = 28.760; df = 12, 156; P < 0.001) significantly affected the egg production per female, while the interaction between those factors did not present statistically significantly differences (F = 1.306; df = 36, 156; P = 0.136) (Table 1).

**Fig. 4:**
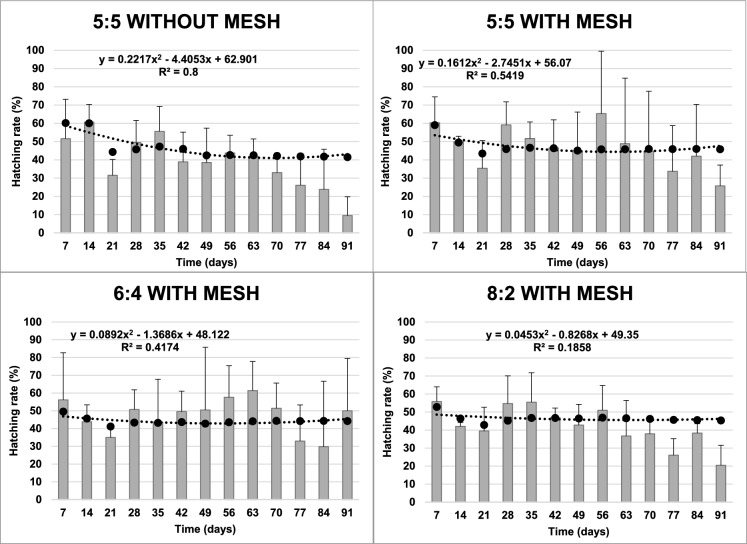
Egg hatching rate of *Zophobas morio* under different adult sex ratio treatments over 91 days (%, mean ± SE). The bars illustrate the hatching rate per female at each time period. The dash lines indicate the fit curve for the cumulative hatching rate per female over time

No significant differences were recorded in terms of cumulative hatching rate at the completion of the experiment among the four different treatments (F = 1.082; df = 3; P = 0.394) with rates ranging from 41.47 to 45.82% (Fig. 4). However, fluctuations were detected during the entire period conducting the experiment regarding the hatching rate. For the 5:5 and 6:4 with mesh treatments the hatching rate ranged from 25 to 65% and 30 to 61%, respectively. The hatching rate of the 8:2 treatment fluctuated between 20 and 56% over time. Nevertheless, the widest range were detected for the control treatment (5:5 sex ratio without mesh) which ranged between 9 and 61% hatching rate, over time. These observations are in accordance with the linear mixed effect model analysis which showed that there were statistically significant differences in hatching rate over time (F = 5.658; df = 12, 156; P < 0.001), while there were no statistically significant differences in terms of sex ratio (F = 2.420; df = 3, 156; P = 0.068) and the interaction (F = 0.551; df = 36, 156; P = 0.981) of the two factors tested (sex ratio and time) regarding the hatching rate.

## Discussion

The reproduction of sexually reproducing animal species is significantly influenced by the adult sex ratio, a critical factor in the evolutionary dynamics of species (Girondot and Pieau 1993; Dyson and Hurst 2004). Fisher’s principle suggests a 1:1 female to male ratio as the evolutionary stable strategy. Most of the scientific studies in edible insects use the equilibrium ratio. However, several insect species deviate from this equilibrium due to factors such as reproductive costs, fitness gain and environmental predictability (West et al. 2002). In the case of *Z. morio,* the optimum adult sex ratio has yet to be explored. Hence, the aim of our study was to investigate how varying female to male ratios affect the reproductive output and longevity of *Z. morio*.

The reproductive dynamics of adults play a crucial role in the survival and biology of any species, as these dynamics directly influence the number of eggs laid and offspring produced. This is particularly affected by the female to male adult ratio (Frooninckx et al. 2022; Rodríguez-Muñoz et al. 2019). Our results show statistically significant differences in the cumulative number of eggs laid per female, with the highest egg production recorded in the control treatment. Interestingly, this did not significantly differ from the 5:5 with mesh treatment, suggesting that the presence of mesh does not have a substantial impact on egg-laying in *Z. morio* rearing. Moreover, we found that females oviposited the highest number of eggs in the third week, after which egg production declined. This pattern of initial peak followed by a gradual decline is consistent with findings from related species such as *T. molitor*, where Morales-Ramos et al. (2012) observed a peak in egg production in the second week of their experiment. Similar findings were reported by Berggreen et al. (2018) and Frooninckx et al. (2022) for *T. molitor*. In contrast, Adamaki-Sotiraki et al. (2022b) found that reproductive allocation varied significantly across *T. molitor* strains from different geographical regions, with each strain exhibiting unique timings for peak egg production. However, comprehensive data on the reproductive output of *Z. morio* with respect to adult sex ratio remain limited.

Interestingly, unlike the egg-laying pattern seen in *T. molitor*, where production decreased over time after the second week (Berggreen et al. 2018; Frooninckx et al. 2022), our results revealed a secondary increase in egg production on day 49 in three treatments (5:5 with and without mesh, as well as 6:4 sex ratios), while the 8:2 sex ratio did not follow this pattern. Several hypotheses may explain this phenomenon. First, changes in reproductive patterns over time could be linked to physiological changes in females (Weil et al. 2006). This is due to the limited resources—such as energy, time and nutrients—that animals allocate to maximize their survival and reproductive success. Second, differential survival rates across the population may influence reproductive dynamics (Service 2000; Cam et al. 2002). Lastly, the terminal investment hypothesis suggests that animals increase their reproductive effort as they age while the possibility of survival decreases (Clutton-Brock 1984; Williams 1966). The observed increase in egg production on the 49th day may align with the last theory, as female survival rates started to decline shortly thereafter, specifically the 56th day of the experiment. This suggests that the optimal reproductive period for *Z. morio* may last approximately 56 days, a useful insight for optimizing rearing practices.

While multiple matings are common across many insect species (Drnevich et al. 2001), mating patterns and female receptivity are still poorly understood (Drnevich 2003). In our study, the hatching rate showed fluctuations over time, with the highest percentage observed in the 5:5 with mesh sex ratio treatment (65%). However, no significant differences in the cumulative number of larvae per female were detected among treatments. For another insect species of the family Tenebrionidae, Jehan et al. (2020) also observed that for *T. molitor* the highest fertility was reached in the balanced adult’s ratio (50% females–50% males). Furthermore, Jehan et al. (2020) noted that females in balanced sex ratio populations produced more offsprings during the first two weeks of adulthood, however females in biased sex ratio populations (75% females and 25% males) maintained reproductive effort for a longer time. The effect of sex ratio on the fecundity of adults has also been tested in other species. More specifically, in a study conducted by Guerra et al. (1972), it was revealed that fecundity was higher for female-biased sex ratio for the insect species *Heliothis virescens* (F.) (Lepidoptera: Noctuidae). On the contrary, Gou et al. (2019) observed higher fecundity in a male-biased population of *Bradysia difformis* Frey (Diptera: Sciaridae). Based on the aforementioned studies, it is apparent that the relationship between sex ratio and fecundity is highly species specific.

It is important to note that, in our study, the hatching rate exhibited a distinct pattern, characterized by a high rate during the first week, followed by a noticeable decline over the subsequent two weeks. This could be explained by the fact that females reduce the number of fertile eggs or terminate the egg laying as sperm in their spermatheca becomes depleted (Ridley 1988). Thornhill and Alcock (1983) argued that some insect females have developed a circle pattern of matings to ensure a consistent supply of sperm and to receive the benefits of multiple matings. While sperm can remain viable in a female’s body for months, maintaining its viability is highly energetically costly (Graham-Smith 1938; Davey and Webster 1967). Consequently, females may choose to mate with multiple males, benefiting from both sperm replenishment and the nutritional value of sperm, which results in a hatching pattern similar to that observed in our study (Ridley 1988). Another reason of this pattern lies in the two key advantages that multiple matings offer to female insects: material and genetic benefits. Material benefits include nutritional resources provided by males to females, such as the fluid included in spermatophores (Gwynne 1997; Eberhard 1996), as well as replenishment of insufficient sperm (Thornhill and Alcock 1983). On the other hand, genetic benefits include the manipulation of offspring paternity (Ridley 1988) and the opportunity to secure genetically superior and compatible sperm (Zeh and Zeh 1996). While the benefits of multiple mating and sex ratio effects on offspring production have been extensively studied in many insect species, they remain underexplored in *Z. morio*. According to our results, however, and evidence from other studies on Tenebrionidae species multiple matings can increase female fertility (Ridley 1988).

Sex ratio plays a crucial role in the cost of reproduction, which is highly sex specific, due to their differing reproductive strategies (Adler and Bonduriansky 2011). In our experiment, the survival rate of adult females varied across the different treatments, with the 8:2 sex ratio treatment presenting the highest final survival rate, which was 75%. Across all treatments, female survival remained above 90% until the 56th day, after which a decline was recorded. The final survival rate ranged from 50% in the control treatment (5:5 without mesh) to 75% in the 8:2 treatment. According to the theory of terminal investment, the present reproduction may reduce future survival and reproductive potential (Clutton-Brock 1984; Williams 1966). This theory may help explain the observed decline in female survival after the 56th day, which was one week following the second peak in egg production. The lowest final survival rate was observed in the control treatment, indicating that sex ratio has a significant impact on female longevity. In particular, mating cost for females is often the result of physical injuries from males, the energy demands of offspring’s reproduction, male harassment and exposure to parasites (Stockley 1997). A study by Jehan et al. (2020) on *T. molitor* found that female mortality was higher in a male-biased population compared to a female-biased ones. This aligns with our results, which showed high survival rate for females in both balanced and female-biased sex ratio treatments. Furthermore, the nutritional benefits provided by males through spermatophores may compensate for some of these costs (Gwynne 1997). Spermatophores are nutrient rich and contribute to somatic maintenance in females (Carver et al. 1999), which can enhance their longevity as demonstrated by several studies in other insect species (Wedell 1996; Lee et al. 2014; Bissoondath and Wiklund 1995).

In contrast, male longevity exhibited a notable decline by the 28th day across all treatments, resulting in a relatively low final survival rate ranging from 35% (5:5 with mesh treatment) to 50% (8:2 treatment). Although there were no statistically significant differences in the male final survival rate among treatments, the more rapid decline occurred in the 5:5 with mesh treatment, starting on day 21 and continuing throughout the experiment. In Tenebrionidae species, each copulation is energetically costly for males, as they transfer their spermatophores containing both sperm and nutrient rich seminal fluid to females (Carver et al. 1999). Producing this seminal fluid, which contains vital proteins, requires significant energy expenditure (Carver et al. 1999). This could be the reason of the reduction in survival rate of males in our experiment after the first peak in reproduction. The disposable soma theory postulates that energy invested in reproduction comes at the cost of body maintenance, leading to gradual declines in both survival and reproduction capacity (Kirkwood 1977). Furthermore, in polyandrous species, like *Z. morio*, males often engage in intense competition for reproductive success, which may include sperm competition, physical combat, or elaborate displays to attract females (Andersson 1994). Such competition incurs considerable energetic and fitness costs, leading to longevity and reduced reproduction (Lane et al. 2010; Hunt et al. 2004; Rodríguez-Muñoz et al. 2019). In line with these findings, Jehan et al. (2020) demonstrated that male longevity was higher in male-biased sex ratios compared to balanced or female-biased populations in *T. molitor*. Our results similarly indicate that as the number of males decreases relative to females, their survival also declines, likely due to the increased number of mating events each male participates in.

## Conclusions

This study investigates the impact of adult sex ratios on the reproductive output and aging costs of *Zophobas morio*. While egg production varied significantly between sex ratios, both balanced and female-biased groups produced sufficient offspring. A marked decline in fecundity and females’ survival after day 56 suggests that replacing adults at this point could improve productivity on an industrial scale. Final survival rates did not differ significantly among treatments, and both balanced and female-biased ratios maximum offspring production. Future research should investigate the mechanisms of reproduction and the effects of aging on fecundity to further enhance large-scale farming practices for this promising species.

## Data Availability

The datasets generated during and/or analysed during the current study are available from the corresponding author on reasonable request.
